# Functionality of cow milk naturally enriched with polyunsaturated fatty acids and polyphenols in diets for diabetic rats

**DOI:** 10.1371/journal.pone.0195839

**Published:** 2018-04-13

**Authors:** Emerson H. Yoshimura, Nadine W. Santos, Erica Machado, Bruna C. Agustinho, Lucelia M. Pereira, Sílvia C. de Aguiar, Anacharis B. Sá-Nakanishi, Cecília E. Mareze-da-Costa, Lucia M. Zeoula

**Affiliations:** 1 Departamento de Zootecnia, Universidade Estadual de Maringá, Maringá, Brazil; 2 Departamento de Zootecnia, Universidade do Estado de Mato Grosso, Pontes e Lacerda, Brazil; 3 Departamento de Bioquímica, Universidade Estadual de Maringá, Maringá, Brazil; 4 Departamento de Ciências Fisiológicas, Universidade Estadual de Maringá, Maringá, Brazil; University of Illinois, UNITED STATES

## Abstract

The increasing incidence of diabetes mellitus is becoming a serious threat to human health in various parts of the world. Studies with dairy products have shown a potential beneficial effect against diabetes. This experiment evaluated the supplementation of milk naturally enriched with polyunsaturated fatty acids (PUFA) and polyphenols in rats with streptozotocin-induced diabetes. Forty male 28-day-old *Wistar* rats were distributed in four experimental treatments of diabetic animals (streptozotocin induction) and a normal group (non-induced). Experimental treatments were: control (water), whole common milk (COM-M), milk enriched with PUFA (PUFA-M), milk enriched with PUFA and polyphenols (PUFA/P-M) through a special diet offered to dairy cows. Milk supplementation at a dose 5 mL/kg body weight was performed for 77 days, 42 days before and 35 days after diabetes induction. The COM-M supplementation increased brown fat deposits, reduced post-induction glucose levels, reduced blood fructosamine levels, and improved glucose tolerance. Milk enriched with PUFA reduced final fasting glucose, LDL levels, and improved blood antioxidant capacity. Milk enriched with PUFA and polyphenols promoted an increase in gastrocnemius muscle mass, and a reduction in mesenteric fat and LDL levels. Milk intake, with an emphasis on milk enriched with PUFA and polyphenols, attenuated the metabolic disorders of streptozotocin-induced diabetes in rats.

## Introduction

Diabetes mellitus (DM) is a metabolic and chronic disease with multifactorial etiology, caused by defects in insulin secretion and/or action and presenting hyperglycemia as a common manifestation [1]. Multiple metabolic disorders, including impaired lipid and lipoprotein metabolism, oxidative stress, subclinical inflammation, and vascular endothelial dysfunction, are common in DM [2]. These long-term changes may result in the occurrence of diseases (DM complications) such as retinopathy [3], nephropathy [4], vascular diseases [5], as well as joint and bone diseases [6], characterizing DM as a syndrome of high morbidity and mortality.

The number of people affected by diabetes is increasing, and global estimates point to more than 430 million individuals in 2030 [7]. The treatment of DM is complex and consists of special diets, physical activity, and control of hyperglycemia [6]. High numbers of diabetics and difficult treatment have stimulated the search for functional foods that help to prevent or treat DM. The concept of functional food has expanded rapidly; in addition to basic nutritional functions, it has potential benefits to promote health and reduce the risk of chronic diseases [8].

The consumption of dairy products has been associated with a reduction of the DM risk and an improvement in metabolic health [9]. In a study with human subjects, the intake of cheese and fermented dairy products reduced glycemic levels in diabetic patients [10]. Milk intake had a significant negative correlation with metabolic syndrome in middle-aged men in a UK study [11], a condition deeply related to diabetes. The beneficial effects of milk have been related to its components such as calcium [12] and whey proteins [13].

Recent studies emphasize the properties of bioactive compounds in functional foods, such as polyunsaturated fatty acids (PUFAs), especially omega-3 fatty acids (FA-n3), which have been correlated with a reduced risk of diabetes and improved human health [14–16]. Differentiated diets for dairy cows with polyunsaturated fat (n3) and polyphenols can naturally enrich milk with PUFA and polyphenols [17]. In a recent study, the administration of this enriched milk as a supplement to obese rats has resulted in increased muscle mass and reduced LDL values [18].

In this experiment, diabetic rats received supplementation with milk naturally enriched with PUFA and polyphenols, obtained by manipulation of dairy cow diet [17,18] to test the hypothesis that enriched cow milk could be a functional food, and facilitate the prevention and treatment of diabetes. Thus, the objective of this study was to determine the effects of such supplementation on feed intake, growth, blood parameters, glucose tolerance and body composition.

## Material and methods

### Animals, treatments and experimental procedures

This experiment was approved by the Ethics Committee for the Use of Animals in Experiments of the State University of Maringá (Maringá, Paraná, Brazil), statement number 115/2012. The experiment was performed in the Physiological Science Department, using animals from the colony of the central animal house of the State University of Maringá. Male *Wistar* rats (*Rattus norvegicus*) were kept into collective cages (46 × 24 × 20 cm), with four animals per cage, under the following room conditions: 24°C, 12-hour light-dark cycle, water and feed provided *ad libitum*. Rats were fed a standard chow diet (Nuvilab CR1, Nuvital, Colombo, Paraná, Brazil) composed of 879.7 g/kg dry matter (DM), 261.6 g/kg DM of crude protein, 22.9 g/kg DM of ether extract, 41.8 g/kg DM of fiber, 972.2 g/kg DM of organic matter and 3,959 kcal/kg DM of gross energy.

Forty 28-day-old rats were distributed into the experimental groups based on body weight (BW) after weaning. Four experimental treatments (*n* = 8 each) with diabetic animals were established: 1) control (water), 2) whole common milk (COM-M), 3) milk enriched with PUFA (PUFA-M), and 4) milk enriched with PUFA and polyphenols (PUFA/P-M). A group of eight rats was maintained under the same conditions under the control group but without diabetes induction to allow comparison of parameters related to diabetes.

Supplementation with milk started after weaning the animals (28 days old) and was performed daily by gavage at 09:00 am. The milk dose was established following the recommendations of the Brazilian Ministry of Health [19], namely 150 liters per year of milk and dairy products for an average weight Brazilian man [20], at 5 mL/kg body weight. The dose was adjusted weekly according to the weight of the animals.

The three types of milk (Table 1) were obtained from dairy cows used in a previous experiment [17] as follows: COM-M was from cows which received a control diet; PUFA-M was from cows fed a diet containing flaxseed oil, rich in omega-3 (25 g/kg DM); PUFA/P-M was from cows fed a diet containing flaxseed oil, a propolis-based product (1.2 g/kg DM) and vitamin E (375 IU/kg DM). After the last milking of each collection day, milk samples were stored in polyethylene tubes (12 mL) and frozen (-20°C). After chemical characterization, PUFA/P-M sample was chosen considering polyphenol content around 19.0 GAE mg/L [17], PUFA-M and COM-M milk were randomly chosen within each treatment. Immediately prior to providing milk to rats, it was thawed (4°C) and vortexed for 5 minutes.

**Table 1:** Composition of common milk (COM-M), milk enriched with polyunsaturated fatty acids (PUFA-M), and milk enriched with PUFA and polyphenols (PUFA/P-M)^1^.

Component	Milk			P
	COM-M	PUFA-M	PUFA/P-M	
Chemical composition (mg/mL)				
Fat	29.80 ± 0.1	26.86 ± 0.8	27.78 ± 0.4	0.18
Protein	28.99 ± 0.2	30.74 ± 0.9	29.26 ± 0.6	0.68
Lactose	46.31 ± 0.1	45.81 ± 0.6	47.50 ± 0.2	0.34
Total solids	114.40 ± 0.4	112.59 ± 0.7	113.95 ± 0.8	0.72
Fatty acid composition (mg/mL)				
12:0	0.82 ± 0.04	0.64 ± 0.14	0.67 ± 0.02	0.87
14:0	2.70 ± 0.08	2.53 ± 0.31	2.49 ± 0.01	0.97
16:0	7.02 ± 0.63	5.96 ± 0.01	5.34 ± 0.12	0.52
18:0	1.73 ± 0.48	3.34 ± 0.19	3.28 ± 0.02	0.11
*trans-*9-18:1	0.18 ± 0.10b	0.58 ± 0.09ab	0.70 ± 0.02a	0.03
18:1 (n-9)	4.01 ± 0.77	6.52 ± 0.55	5.89 ± 0.21	0.10
18:2 (n-6)	0.47 ± 0.16	0.66 ± 0.05	0.70 ± 0.01	0.15
18:3 (n-3)	0.06 ± 0.02b	0.30 ± 0.02a	0.29 ± 0.01a	0.004
*cis-*9,*trans-*11-18:2	0.09 ± 0.03	0.12 ± 0.01	0.12 ± 0.01	0.12
*trans-*10,*cis-*12-18:2	0.02 ± 0.01	0.03 ±0.01	0.03 ±0.01	0.35
Total CLA	0.11 ± 0.03	0.15 ± 0.01	0.15 ± 0.01	0.14
SFA	13.98 ± 4.34	14.00 ± 0.43	13.42 ± 0.65	0.98
MUFA	5.43 ± 1.17	8.23 ± 0.52	7.61 ± 0.17	0.14
PUFA	0.67 ± 0.22b	1.14 ± 0.09a	1.17 ± 0.02a	0.04
n-6/n-3	8.61a	2.28b	2.55b	<0.001
Antioxidant quality and oxidative stability				
Total polyphenols (GAE mg/L)^2^	11.33 ± 0.61b	10.16 ± 0.69b	18.15 ± 1.30a	0.03
Orac (TE mmol/L)^3^	10.57 ± 0.29	11.18 ± 0.70	14.70 ± 0.06	0.56
Reducing power (GAE mg/L)	25.85 ± 0.32	35.78 ± 0.57	38.32 ± 0.59	0.05
Conjugated diene (mmol/kg fat)	41.51 ± 0.09b	59.55 ± 0.28a	54.53 ± 0.53ab	0.03
Tbars (MDAE mmol/kg fat)^4^	2.68 ± 0.34	5.28 ± 0.76	3.43 ± 0.89	0.52

^1^Milk composition reported by Santos et al. [18]. Data are mean ± standard deviation from a single sample for each treatment. CLA = conjugated linoleic acid, SFA = saturated fatty acids, MUFA = monounsaturated fatty acids. Means with different letters differ by Tukey's test.

^2^GAE = gallic acid equivalent

^3^TE = Trolox® equivalent

^4^MDAE = malondialdehyde equivalent.

The total experimental period was 77 days, beginning when animals were 28 days old and diabetes induction was performed after 42 experimental days, when the animals were 70 days old. Milk supplementation continued for 35 days until the animals were 105 days old when they were euthanized.

To induce experimental diabetes, animals in 12-hour overnight fast received a single intravenous injection (penile vein) of streptozotocin (Streptozotocin®, Sigma, St Louis, MO, United States), dose 40 mg/kg (BW), dissolved in citrate buffer (0.05 M, pH 4.5). Animals remained fasted for four hours after application of the diabetogenic drug.

After seven days of diabetes induction rats were placed in individual metabolic cages to measure feed and water intake, and urine production. The metabolic cages contained a drinking bottle with volumetric graduation, allowing the recording of water intake. These cages presented the floor full of small holes coupled to a collecting funnel that allowed the collection of the urine produced in bottles located below the cage. A previous 24 hours were given for adaptation and the followed 24 hours for measuring parameters. Data from normal rats (*n* = 8) were used to verify the success of diabetes induction.

Animals were weighed weekly and in the last week of the experimental period, after 12 h of fasting, rats were submitted to the oral glucose tolerance test (1 g/kg BW). Blood samples were collected by caudal puncture at 0, 30, 60, 90, and 120 minutes and glucose was determined on a glycosimeter MediSense® Optium™ (Abbott Diabetes Care Inc., Alameda, CA, United States).

At the end of the experimental period, after 12 h of fasting, the animals were anesthetized with sodium thionembutal (Thionembutal, Abbott, São Paulo, SP, Brazil) at 40 mg/kg body weight. After recording the naso-anal length, the animals were submitted to median laparotomy and blood samples were collected through the inferior vena cava. Blood samples were centrifuged (2,500 *g*, 20 min) and stored (-20°C) for further analysis. Four deposits of white adipose tissue were dissected and weighed: periepididimal, retroperitoneal, mesenteric and subcutaneous fats. The periepididimal fat was obtained by a horizontal cut above the epididymus. The retroperitoneal fat was obtained by first separating the perirenal fat, and then dissecting the retroperitoneal pad. The mesenteric fat was accessed by cross-sectioning the intestine at the duodenal-jejunum junction, and then stripping the fat from the intestinal loops carefully. The subcutaneous inguinal was accessed by dissecting all the fat in the inguinal region to a horizontal line parallel to the xiphoid cartilage [21].

The interscapular brown adipose tissue, which represents the largest brown fat deposit in the rat [22] was also dissected and weighed. Liver, testicles, seminal vesicles, kidneys, soleus and gastrocnemius muscles, and periepididymal, retroperitoneal, mesenteric, subcutaneous, and brown fat deposits were removed and weighed. A liver fragment was frozen in liquid nitrogen and maintained at -80°C. Liver fat content was determined according to Folch et al. [23]. The Lee index was obtained by the ratio between the cube root of the body weight and the naso-anal length [24].

### Chemical analysis

Determination of milk chemical composition, fatty acid composition, and antioxidant composition was described by Santos et al. [18]. Determination of blood concentrations of glucose, fructosamine, total cholesterol, HDL cholesterol, triacylglycerol, aspartate aminotransferase, and alanine aminotransferase were determined using colorimetric methods (Gold Analisa^*®*^, Belo Horizonte, MG, Brazil) and measured on a spectrophotometer (Bioplus2000^®^, São Paulo, SP, Brazil). The LDL cholesterol concentration was estimated by the Friedewald equation: LDL cholesterol (mg/100 mL) = total cholesterol–HDL–(triacylglycerol/2.2).

Oxidation of blood proteins was evaluated by determination of reduced thiols [25]. Total antioxidant capacity (TAC) of blood was analyzed by using ABTS radical (2,2'-azino-bis[3-ethylbenzothiazoline-6-sulphonic acid]) [26].

### Statistical analysis

Milk composition was analyzed by variance test and Tukey’s test in order to characterize the milk types. Data from non-diabetes-induced rats were analyzed from those of diabetes-induced rats by variance test and Tukey’s test to demonstrate the efficiency of diabetes induction. The experimental design was completely randomized. Other variables were analyzed in a variance test according to the following model: Y_*ijk*_ = μ + A_*i*_ + T_*j*_ + C_*k*_ + e_*ijk*_, where Y_*ijk*_ = dependent variables, μ = overall mean; A_*i*_ *=* effect of animal (*i* = 1–8); T_*i*_ = effect of treatment (*i* = 1–4); C_*j*_ = effect of cage (*i* = 1–2); e_*ij*_ = random residual error. Orthogonal contrasts were used to compare effects between groups: 1) milk supplementation (control vs. COM-M, PUFA-M and PUFA/P-M); 2) supplementation with PUFA-milk (COM-M vs. PUFA-M and PUFA/P-M); and 3) supplementation with PUFA/P-milk (PUFA-M vs. PUFA/P-M). Analyses of glucose values obtained during glucose tolerance test were performed as repeated measures in the MIXED procedure of SAS 9.0, considering the effects of animal, treatment, time, and treatment*time interaction in the model. Values of area under the curve were determined by the trapezoidal rule using GraphPad Prism® software, version 5.0, considering fasting glucose level for the baseline. Significance was declared at *P* < 0.05, and a tendency to be significant was accepted at *P <* 0.10.

## Results

Fasting hyperglycemia (324.6 ± 54.6 *vs* 85.1 ± 11.8 mg/100 mL; *P* < 0.001), lower body weight (263.5 ± 33.2 *vs* 364.8 ± 28.6 g; *P* < 0.001), polydipsia (91.0 ± 13.0 *vs* 49.5 ± 3.8 mL/day; *P* < 0.001), and polyuria (64.6 ± 6.3 *vs* 9.4 ± 4.1 mL/day; *P* < 0.001) in diabetic rats, when compared to normal rats, respectively, confirmed the establishment of the pathophysiological condition that is characteristic of the diabetes model induced by streptozotocin. Milk supplementation immediately after weaning had acceptability by diabetic rats without any diarrhea events during the experimental period.

Feeding dairy cows with a diet containing a high FA n-3 content (flaxseed oil) provides naturally enriched milk (PUFA-M and PUFA/P-M) with a higher proportion of FA n-3 than common milk (*P* = 0.004, Table 1). Adding antioxidant compounds (propolis extract and vitamin E) to the same PUFA diet for dairy cows increased antioxidant quality in milk as the total polyphenol increased in PUFA/P-M compared to PUFA-M (*P* = 0.03).

Orthogonal contrasts were used to analyze the effects of experimental groups, as previously described. Contrast 1 isolated the effect of milk supplementation (COM-M, PUFA-M, and PUFA/P-M against the control) and is cited along the text as milk effect or milk supplementation. Contrast 2 isolated the effect of milk enriched with PUFA (PUFA-M and PUFA/P-M). Contrast 3 isolated the effect of milk enriched with PUFA and polyphenols (PUFA/P-M). The analyzed variables were not affected by cage effect.

Milk supplementation to the diet of diabetic rats did not alter (*P* > 0.05) final body weight, naso-anal length, Lee index, the water and feed intake, the urine production and the weight of liver, soleus muscle, and periepididimal and subcutaneous fat deposits, and liver fat content when compared to the control group (Table 2).

**Table 2:** Body weight (BW), naso-anal length, Lee index, water and feed intake, urine production, and organ and fatty tissue weights relative to body weight of control diabetic rats (control) and diabetic rats fed diets supplemented with common milk (COM-M), milk enriched with polyunsaturated fatty acids (PUFA-M), or milk enriched with PUFA and polyphenols (PUFA/P-M).

Parameters	Treatment				P^1^		
	Control	COM-M	PUFA-M	PUFA/P-M	1	2	3
Initial body weight (g)	84.50 ± 15.09	82.43 ± 11.56	84.10 ± 17.50	83.61 ± 12.07	0.61	0.84	0.92
Final body weight (g)	256.75 ± 31.53	259.14 ± 32.75	273.10 ± 36.28	265.22 ± 37.56	0.47	0.54	0.67
Naso-anal length (cm)	21.40 ± 1.02	21.57 ± 0.67	21.40 ± 0.65	21.11 ± 0.93	0.88	0.45	0.57
Lee index ^2^	304.98 ± 9.13	305.88 ± 6.08	309.47± 11.23	307.32 ± 6.53	0.40	0.52	0.63
Feed intake (g/day)^3^	33.80 ± 4.04	34.50 ± 2.45	35.80 ± 6.72	33.30 ± 4.04	0.78	0.98	0.45
Water intake (mL/day)^3^	90.00 ± 14.14	92.00 ± 10.37	93.00 ± 12.04	89.00 ± 18.51	0.86	0.90	0.66
Urine production (mL/day)^3^	63.60 ± 5.41	62.82 ± 6.64	65.84 ± 7.75	66.00 ± 6.63	0.71	0.41	0.97
Liver (g/100g BW)	4.43 ± 0.27	4.33 ± 0.36	4.38 ± 0.29	4.19 ± 0.36	0.32	0.77	0.28
Gastrocnemius muscle (g/100g BW)	0.45 ± 0.10	0.40 ± 0.06	0.42 ± 0.05	0.52 ± 0.06	0.97	0.03	0.01
Soleus muscle (g/100g BW)	0.05 ± 0.01	0.05 ± 0.01	0.04 ± 0.00	0.04 ± 0.01	0.29	0.65	0.73
Liver fat (g/100g BW)	2.90 ± 0.23	2.97 ± 0.12	2.93 ± 0.23	2.92 ± 0.24	0.70	0.66	0.95
Periepididymal fat (g/100g BW)	0.44 ± 0.15	0.58 ± 0.24	0.44 ± 0.18	0.41 ± 0.25	0.60	0.13	0.83
Retroperitoneal fat (g/100g BW)	0.12 ± 0.12	0.28 ± 0.32	0.22 ± 0.21	0.20 ± 0.35	0.25	0.60	0.91
Subcutaneous fat (g/100g BW)	0.30 ± 0.17	0.37 ± 0.23	0.24 ± 0.09	0.34 ± 0.19	0.69	0.37	0.38
Brown fat (g/100g BW)	0.03 ± 0.02	0.04 ± 0.01	0.05 ± 0.01	0.05 ± 0.01	0.005	0.50	0.53
Mesenteric fat (g/100g BW)	0.29 ± 0.12	0.31 ± 0.08	0.35 ± 0.03	0.21 ± 0.04	0.98	0.57	0.027

Data are mean ± standard deviation.

^1^Probability of significant orthogonal contrasts. Effects tested using orthogonal contrasts were between: 1) Control vs. COM-M, PUFA-M, and PUFA/P-M; 2) COM-M vs. PUFA-M and PUFA/P-M; and 3) PUFA-M vs. PUFA/P-M.

^2^Ratio between cubic root of body weight and naso-anal length.

^3^After seven days of diabetes induction.

There was a significant positive effect of milk supplementation (COM-M, PUFA-M, and PUFA/P-M) on the amount of interscapular brown adipose tissue (*P* = 0.005) compared to the control (Table 2). The PUFA/P-M supplementation increased gastrocnemius muscle weight (*P* = 0.01) and reduced mesenteric fat (*P* = 0.027) compared to PUFA-M. Mean values of deposits of retroperitoneal adipose tissue were 2.0-fold higher in animals receiving milk supplementation. Although such differences were not statistically significant, they should be noted.

**Fig 1:**
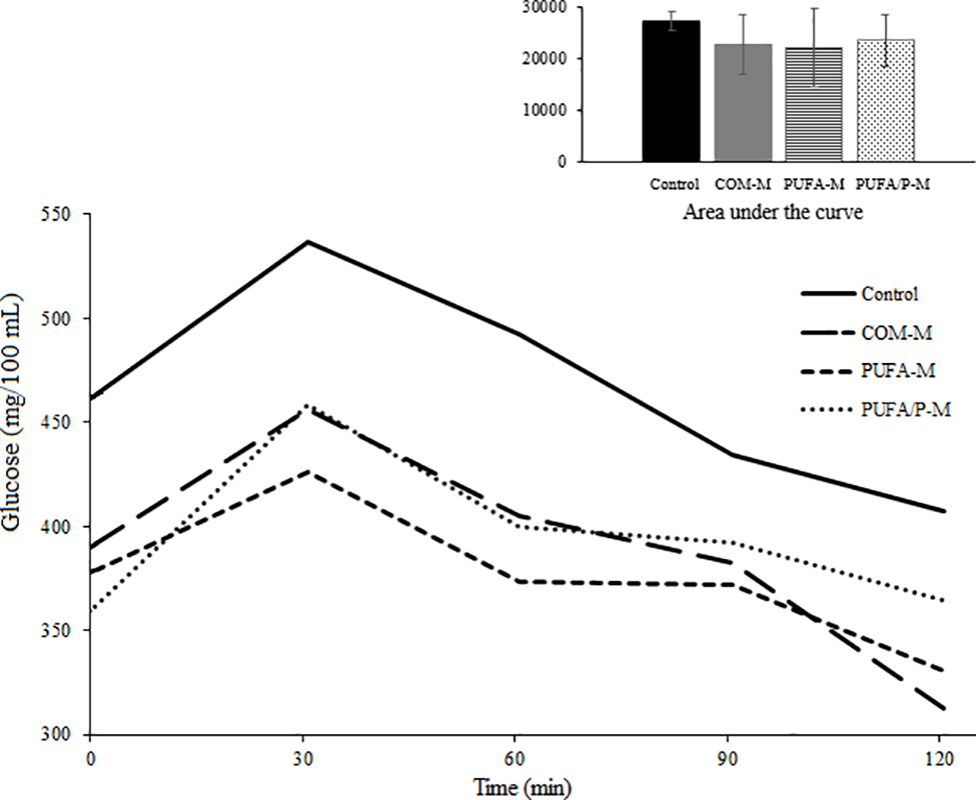
Blood glucose collected every 30 minutes from 0 to 120 minutes after glucose intake in glucose tolerance test in diabetic rats. Control = water, COM-M = common milk, PUFA-M = milk enriched with polyunsaturated fatty acids, PUFA/P-M = milk enriched with PUFA and polyphenols. SEM = 11.9; treatment = *P* = 0.0003; time = *P* = 0.0008; treatment x time = *P* = 0.99. Orthogonal contrasts of mean glucose level: *P*1 < 0.0001, *P*2 = 0.78, *P*3 = 0.44. Orthogonal contrasts of area under the curve: *P*1 = 0.047, *P*2 = 0.95, *P*3 = 0.60 (detail); effects tested were: P1 = Control vs. COM-M, PUFA-M, and PUFA/P-M; P2 = COM-M vs. PUFA-M and PUFA/P-M; and P3 = PUFA-M vs. PUFA/P-M.

Glucose levels, measured in a thirty minutes basis (Fig 1), in the four treatments had similar behavior regardless of time after glucose intake (treatment x time *P* = 0.99). There was significant effect of milk supplementation (*P* = 0.0003) on glucose levels, and the contrast analysis showed that COM-M supplementation resulted lower mean levels of glucose along the time (*P* = 0.0001). Between PUFA-M and PUFA/P-M supplementation, similar glucose levels were observed. Values of area under the curve (Fig 1, detail) confirmed the milk effect (COM-M) in improving glucose tolerance (*P* = 0.047), without effect of PUFA-M and PUFA/P-M supplementation.

Rats which received milk presented a milder hyperglycemic condition (fasting glucose) after diabetes inducing (*P* = 0.02) (Table 3). Milk supplementation did not alter blood levels of triacylglycerol, total cholesterol and cholesterol fractions, aspartate transaminase, alanine aminotransferase, and thiols compared to the control; however, fructosamine levels were lower (*P* = 0.01) and total antioxidant capacity were elevated (*P* = 0.03). Rats which received PUFA-M had lower levels of LDL (*P* = 0.01) and superior antioxidant capacity (*P* < 0.0001) compared to those which received COM-M. The PUFA/P-M supplementation resulted in a decrease in LDL (*P* = 0.01) compared to PUFA-M supplementation, but the fructosamine was higher (*P* = 0.004) than PUFA-M group.

**Table 3:** Plasma biochemical profile of control diabetic rats (control) and diabetic rats fed diets supplemented with common milk enriched with polyunsaturated fatty acids (PUFA-M), or milk enriched with PUFA and polyphenols (PUFA/P-M).

Parameters	Treatment				P^1^		
	Control	COM-M	PUFA-M	PUFA/P-M	1	2	3
Post-induction glucose (mg/100 mL)^2^	328.33 ± 50.00	297.57 ± 58.05	272.60 ± 49.70	257.00 ± 47.06	0.02	0.13	0.51
Final glucose (mg/100 mL)^3^	341.14 ± 53.07	353.20 ± 61.19	303.60 ± 53.84	300.57 ± 46.46	0.41	0.11	0.91
Triacylglycerol (mg/100 mL)	73.76 ± 13.42	75.91 ± 11.03	65.76 ± 14.04	67.39 ± 15.73	0.55	0.26	0.85
Total cholesterol (mg/100 mL)	65.59 ± 8.14	71.41 ± 12.12	65.84 ± 12.72	66.98 ± 7.39	0.55	0.30	0.86
VLDL (mg/100 mL)	14.75 ± 2.68	15.18 ± 2.21	13.15 ± 2.81	13.48 ± 3.15	0.55	0.26	0.85
HDL (mg/100 mL)	38.05 ± 2.90	41.61 ± 5.48	39.57 ± 6.00	44.20 ± 8.67	0.21	0.88	0.23
LDL (mg/100 mL)	12.79 ± 2.79	14.62 ± 1.87	13.12 ± 0.84	9.30 ± 2.87	0.71	0.01	0.01
AST (mg/100 mL)	102.11 ± 20.05	115.58 ± 22.73	100.56 ± 19.33	100.36 ± 19.01	0.72	0.18	0.96
ALT (mg/100 mL)	45.99 ± 7.39	45.51 ± 6.13	48.48 ± 7.47	46.59 ± 8.36	0.91	0.61	0.66
Fructosamine (mg/100 mL)	1.41 ± 0.15	1.14 ± 0.22	1.02 ± 0.29	1.36 ± 0.26	0.01	0.64	0.004
Reduced thiols (nmol/mg protein)	4.06 ± 0.71	4.18 ± 0.55	4.16 ± 0.51	4.21 ± 0.36	0.67	0.99	0.86
TAC (μmoles/mg protein)	4.11 ± 0.93	3.40 ± 0.71	5.66 ± 0.79	5.92 ± 0.64	0.03	<0.0001	0.72

Data are mean ± standard deviation. AST = aspartate transaminase, ALT = alanine aminotransferase, TAC = total antioxidant capacity.

^1^Probability of significant orthogonal contrasts. Effects tested using orthogonal contrasts were between: 1) Control vs. COM-M, PUFA-M, and PUFA/P-M; 2) COM-M vs. PUFA-M and PUFA/P-M; and 3) PUFA-M vs. PUFA/P-M.

^2^ Fasting blood glucose at 24 hours after diabetes induction (animals awake)

^3^Fasting blood glucose on the day of euthanasia (anesthetized animals).

## Discussion

The supplementation with milk (COM-M, PUFA-M and PUFA/P-M) before and after induction of diabetes with streptozotocin, improved metabolic condition in diabetic rats, lowering fasting post-induction glucose and blood fructosamine concentration (Table 3), and improving glucose tolerance (Fig 1).

Diabetes mellitus can produce metabolic decompensations and generate chronic complications at long-term, such as blindness [3,27], atherosclerosis [28,29], renal insufficiency [4], and myocardial infarction [5]. Hyperglycemic control in diabetic patients is essential for the prevention of these associated chronic diseases [30]. Milk supplementation may be an important tool in glucose control in diabetes as observed in this experiment. This result may be corroborated by other studies showing an inverse relationship between intake of dairy products and susceptibility to diabetes [9]. Such effects may be related to calcium or whey proteins in the milk, both with indirect effects on blood glucose. In an experiment with obese rats, calcium supplementation decreased the production of inflammatory cytokines, oxidative stress, and lipid accumulation in fat cells [12]; such reductions may lead to improved insulin sensitivity and a reduction in DM hyperglycemia. Whey proteins can confer glucose regulatory properties by stimulating the secretion of intestinal and incretin hormones which regulate insulin release [13].

Diabetic rats which received milk supplementation had higher brown fat deposition than the rats in the control group (Table 2). Brown fat produces heat depending on lipid metabolism and exhibits a high rate of glucose uptake [31]. This tissue also responds to insulin with a 5-fold increase in glucose uptake, without any change in blood flow [32]. The increase in such tissue may have contributed to the beneficial metabolic effects of milk supplementation.

We also evaluated fructosamine blood levels (Table 3). Fructosamine reflect recent glucose control as there is a direct relationship between hyperglycemic levels and the glycation degree of proteins in the blood. Several functional disorders result from protein glycation, with the main determinants of vascular diseases being associated with DM [33]. In this experiment, milk intake reduced fructosamine levels in diabetic rats, indicating that such animals had better glycemic control than diabetic animals which did not receive milk.

Phenolic compounds of milk are mostly derived from the ruminant diet [34] in the form of metabolites of dietary phenolic compounds modified in the rumen before absorption [35]. The supplementation of dairy cows with a propolis-based product and vitamin E [17] enriched the milk with polyphenols (Table 1), resulting in a positive effect on gastrocnemius muscle mass and a decrease in mesenteric fat mass (Table 2), an improvement in body composition. In DM, the intake of phenolic compounds is beneficial as observed in studies with medicinal plants containing flavonoids as major phenolic compounds [36,37].

In DM, there are significant changes in the concentration, composition, and metabolism of lipids. Changes are of oxidative state, with increased lipid peroxidation associated with hyperlipidemia [36]. Dyslipidemia is an important risk factor for cardiovascular diseases in patients with DM [38], which are two to three times more likely to have a myocardial infarction and stroke [39]. Thus, the control of these parameters is fundamental to prevent complications due to DM. This experiment shows that milk enriched with PUFA and milk enriched with PUFA and polyphenols can reduce LDL in diabetes (Table 3).

Dairy products, when classified according to low or high fat contents, do not always present a linear dose response in diabetes prevention [40,41], demonstrating that other factors, such as fat composition, may also have positive effects. The PUFA-enriched milk presented an n6:n3 ratio of 2.28:1 (Table 1), below the recommended maximum value of 4:1 for health benefits [42]. The beneficial effects of PUFA and fatty acids n3 on human health have been recognized previously [15]. These benefits were evidenced by the regulation in metabolic and inflammatory pathways, cardiovascular diseases, as well as in glucose homeostasis and insulin sensitivity [14,43]. In a meta-analysis of published articles with human subjects, the intake of FA n3 statistically reduced DM2 risk [16].

Other similar studies in the diabetic animal model were not found in the literature [18]. Thus, we could verify that supplementing the diets of dairy cows with PUFA-rich oil and polyphenols transfers part of these compounds into milk. This naturally enriched milk had a functional effect in the prevention and treatment of diabetes mellitus.

## Conclusion

Whole common milk shown to be effective in improving the effects resulting from diabetes. Milk naturally enriched with PUFA and polyphenols in diets may add benefits when considering milk supplementation in a diabetic condition.

Dietary supplementation of diabetic rats with whole common milk increased brown fat deposits, reduced post-induction glucose levels, reduced blood fructosamine levels, and improved glucose tolerance. Milk enriched with PUFA, through a special diet offered to cows, reduced final fasting glucose, LDL levels, and improved blood antioxidant capacity. Milk enriched with PUFA and polyphenols promoted an increase in gastrocnemius muscle mass and a reduction in LDL levels.
